# Measuring chronic care management experience of patients with diabetes: PACIC and PACIC+ validation

**DOI:** 10.5334/ijic.862

**Published:** 2012-10-01

**Authors:** Hanneke W Drewes, Janneke T de Jong-van Til, Jeroen N Struijs, Caroline A Baan, Fetene B Tekle, Bert R Meijboom, G.P Westert

**Affiliations:** Scientific Centre for Care and Welfare (Tranzo), Tilburg University, The Netherlands; and National Institute for Public Health and the Environment, Centre for Prevention and Health Services Research, Bilthoven, The Netherlands; National Institute for Public Health and the Environment, Centre for Prevention and Health Services Research, P.O. Box 1, 3720 BA Bilthoven, The Netherlands; National Institute for Public Health and the Environment, Centre for Prevention and Health Services Research, P.O. Box 1, 3720 BA Bilthoven, The Netherlands; National Institute for Public Health and the Environment, Centre for Prevention and Health Services Research, P.O. Box 1, 3720 BA Bilthoven, The Netherlands; Tilburg School of Social and Behavioral Science, Tilburg University, P.O. Box 90153, 5000 LE Tilburg, The Netherlands; Scientific Centre for Care and Welfare (Tranzo), Tilburg University, P.O. Box 90153, 5000 LE Tilburg, The Netherlands; Scientific Institute for Quality of Healthcare (IQ Healthcare), Radboud University Nijmegen Medical Centre, Postbus 9101, 6500 HB Nijmegen, The Netherlands

**Keywords:** chronic care model, patient experience, chronic care management, integrated care, diabetes, PACIC

## Abstract

**Background:**

The patient assessment of chronic illness care (PACIC) is a promising instrument to evaluate the chronic care experiences of patients, yet additional validation is needed to improve its usefulness.

**Methods:**

A total of 1941 patients with diabetes completed the questionnaire. Reliability coefficients and factor analyses were used to psychometrically test the PACIC and PACIC+ (i.e. PACIC extended with six additional multidisciplinary team functioning items to improve content validity). Intra-class correlations were computed to identify the extent to which variation in scores can be attributed to GP practices.

**Results:**

The PACIC and PACIC+ showed a good psychometric quality (Cronbach’s alpha’s >0.9). Explorative factor analyses showed inconclusive results. Confirmative factor analysis showed that none of the factor structures had an acceptable fit (RMSEA>0.10). In addition, 5.1 to 5.4% of the total variation was identified at the GP practice level.

**Conclusion:**

The PACIC and PACIC+ are reliable instruments to measure the chronic care management experiences of patients. The PACIC+ is preferred because it also includes multidisciplinary coordination and cooperation—one of the central pillars of chronic care management—with good psychometric quality. Previously identified subscales should be used with caution. Both PACIC instruments are useful in identifying GP practice variation.

## Background

Chronic care management for patients with diabetes has changed in recent decades. Initiatives such as multidisciplinary protocols, pro-active care plans, and additional education have been introduced [1, 2]. Most of these initiatives are in line with the widely adopted chronic care model (CCM) [2]. The CCM is promoted as a guide to improve chronic care to realise patient-centred care in which problems such as fragmentation, guideline non-adherence, and restricted self-management are limited [3, 4]. Because patient-centredness is becoming more and more important in chronic care, it is of importance to measure the chronic care experiences of patients [5]. Patients’ chronic care experience is positively related to other aspects of health care quality, including their engagement with and adherence to provider’s instructions as well as clinical processes and outcomes [6]. Moreover, patients’ experiences can be used for quality improvement or even as a benchmark tool [5–9].

Several instruments exist to measure patient’s chronic care experiences [9]. The Patient Assessment of Chronic Illness Care (PACIC), which measures the extent of alignment of chronic care with the CCM, is one of the most promising instruments to measure patients’ chronic care experience [8–11]. Previous studies suggest that PACIC scores can be used to direct quality improvement programs [8–11].

Notwithstanding the promising results from previous studies, additional validation of the PACIC is needed to improve this instrument [9, 11–13]. First, it is still unknown which PACIC subscales are appropriate to use. Previous validation studies, with the exception of the recently performed validation by Gugiu, used inappropriate methodological tests for PACIC’s ordinal data structure [12, 13]. Gugiu validated the PACIC with a modified response scale to avoid an ordinal structure; however, this modified response scale was unsuccessful [12, 13].

Second, the PACIC is assumed to measure the extent to which chronic care, for instance diabetes care, is congruent with the CCM. However, not all components of the CCM are fully taken into account. In particular, the functioning of the multidisciplinary team, i.e. collaboration and coordination, is only briefly mentioned in the PACIC. Additional team-functioning items would increase the content validity because the CCM assumes that interventions on the practice level aim to improve the functioning of the pro-active multidisciplinary team and thereby the quality of chronic care management.

Third, as far as we know, it has not yet been determined if and how the PACIC could be used to compare the quality of chronic care between GP practices. Dutch GP practices, including GPs and GP practice nurses working at the same address, provide diabetes care collaboratively. Patients’ experiences of chronic care management within a GP practice could be useful as a marketing tool [5]; however, it is unknown whether the PACIC identifies differences in patients’ experiences between GP practices. As patients’ perspective ratings on the quality of chronic care could only be reliably interpreted by case-mix adjustment, insight into the influence at the GP practice level and individual characteristics is needed [14, 15].

Although the PACIC is considered to be the most appropriate instrument to measure patients’ chronic care experience [9], several questions need to be answered to improve its usefulness. The objectives of this study are the following: 1) to assess the psychometric quality of the PACIC using the appropriate psychometric tests for ordinal data; 2) to assess the psychometric quality of the PACIC+, that is, the PACIC including six additional multidisciplinary team functioning variables; and 3) to test the ability of the PACIC and PACIC+ to discriminate between GP practices.

## Theory and methods

### Study population

Data were obtained from an observational study evaluating the effects of a bundled payment system for diabetes care in the Netherlands. Details about this study are reported elsewhere [16]. For the observational study, ten different care groups were selected based on size, catchment area, geographical location and composition (e.g. rural vs. urban), and their organisational structure. Care groups are legal entities—formed by multiple care providers often exclusively GPs—which operate as contracting entities to cover a full range of diabetes care services for a fixed period. Care groups can decide to either deliver the various diabetes care components themselves or subcontract other care providers [17]. The characteristics of the included care groups are outlined in Appendix 1.

As part of the observational study, a questionnaire was sent to a random sample of 4377 diabetes patients clustered within a random sample of 78 GP practices representing eight care groups. The people that receive their diabetes care by the GP practices are predominantly people with diabetes type 2. The goal of the questionnaire was to assess the patients’ experiences with chronic care, and it incorporated questions about demographic and clinical patient characteristics, the PACIC+, and patient outcomes. The first three authors sent the questionnaires to the patients. After three weeks, reminders were sent to non-respondents.

### Measures

The PACIC was used to identify the extent to which the chronic care was congruent with Wagner’s CCM in the past 12 months. The PACIC consists of 20 questions with response categories ranging from 1 ‘almost never’ to 5 ‘almost always’, with higher scores indicating a higher extent to which patients received integrated care following the CCM elements [8]. We used the Dutch PACIC translated by Vrijhoef et al. [9]. Glasgow et al. identified five subscales of the PACIC: 1) Patient activation (3 items), 2) Delivery system design/Decision support (3 items), 3) Goal setting (5 items), 4) Problem solving/Contextual counselling (4 items), and 5) Follow-up/Coordination (5 items) [8]. The subscores for each scale were computed by averaging across items completed within that scale, and the overall PACIC was scored by averaging scores across all subscales.

Furthermore, the PACIC was upgraded by including six additional questions regarding multidisciplinary team functioning, i.e. collaboration and coordination, which are used in the Dutch consumer quality index (CQ-index) instrument [18, 19] and the Dutch panel of chronic illnesses [20]. The CQ-index instruments were developed in the Netherlands to assess the quality of care based on the American Consumer Assessment of Health care Providers and Systems (CAHPS) and the Dutch Quality of Care Through the Patient’s Eye (QUOTE) [14, 18]. The scores on these six additional items have identical response categories and scores as the PACIC. The 20 items of the PACIC and the 6 additional items of the PACIC+ are outlined in Appendix 2.

The demographic and clinical patient characteristics included in the study were age, sex, ethnicity, educational level, type of diabetes, duration of diabetes, and co-morbidity. Ethnicity was defined as Western (North-America, EU (except Turkey), Japan, Indonesia) and non-Western, and education was defined as low (lower vocational or less education), middle (secondary education), and high (higher education).

### Analysis

Descriptive analyses were applied to describe the baseline characteristics of our study population. The psychometric quality of the PACIC and PACIC+ was measured by reliability and factor analysis. The reliability was tested by assessing the internal consistencies with the Cronbach’s alpha. A Cronbach’s alpha of 0.80 or higher was accepted as a good score [21]. The factor analysis included an explorative factor analysis (EFA) and confirmatory factor analysis (CFA) using the split-half method. After splitting the data-file randomly, we performed EFA with the first half. Three types of EFA were applied to identify the factor structure. Principal axis factoring (PAF) with oblimin rotation was first performed to explore the factor structure of the data. Subsequently, parallel analysis (PA) was performed to identify the number of factors following the O’Connor’s SPSS syntax, which is suitable for ordinal data. Lastly, Velicer’s MAP test was applied with O’Connor’s syntax to perform a factor analysis based on the polychoric correlation matrix obtained in R [22]. Subsequently, CFA was performed in R with the second half of the data to confirm the hypotheses concerning the underlying structure generated by the EFA as well as the factor structure determined by Glasgow. CFA tests the correlation structure of the data against the hypothesised structure and rates the ‘goodness of fit’ [21, 23]. A value >0.10 of the root-mean-square-error-of-approximation (RMSEA) fit index indicates an unacceptable fit of the model, which implies that the correlation between the items within the tested factor structure might be a coincidence [21].

We tested the ability of the questionnaire to discriminate between GP practices by performing multilevel analysis in SPSS [24] because differences in PACIC scores were expected based on the fact that differences in chronic care management are prevalent [25]. A GP practice was defined as one or more GPs working at the same location. The Intra-Class Correlation (ICC) was computed to identify which part of the total variance in the outcomes could be attributed to the difference between GP practices. We examined the influence of GP practice level before (null model) and after case-mix adjustment (full model).

### Missing value imputation

Complete case analysis was applied because the PACIC is most frequently analysed using complete case analysis [9, 11, 26–28]. However, because multiple imputation offers advantages over complete case analyses [29, 30], we also performed the analysis with multiple imputation (MI) datasets. Missing values were imputed using the mice (multiple imputation by chained equation) procedure in R [31]. The mice procedure assumes that the distribution of each variable given the others can be modelled with a logistic regression if the variable is dichotomous, polytomous logistic regression if it is categorical with three or more categories, or linear regression if it is continuous with predictive mean matching [32]. The Gibbs sampler was used to determine the necessary number of iterations to compute 20 imputations for each missing value [31].

## Results

### Patient characteristics

**Figure 1.**
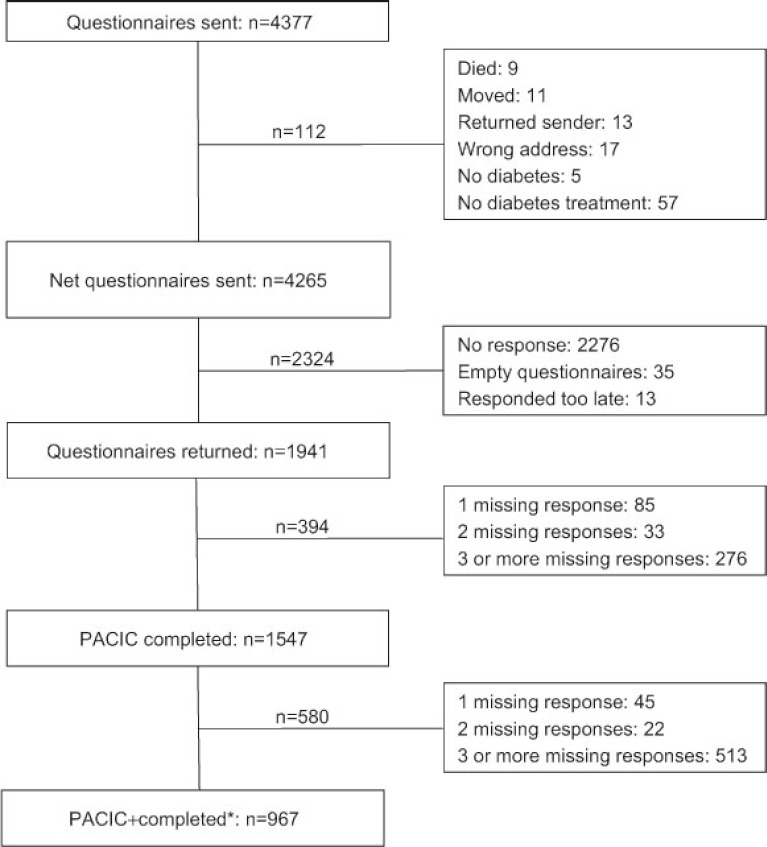
Questionnaire response. *Respondents that completed the PACIC and the 6 additional multidisciplinary team functioning questions of the returned questionnaire.

A total of 1941 patients returned the questionnaire (46% response rate) (Figure 1). Of them, 1547 (80%) completed all PACIC items, while at least one of the items was not completed by the remaining number of patients (20%). In total 967 patients completed the six additional items of the PACIC+.

Respondents that fully completed the PACIC differed from those who had one or more missing response (Appendix 2). Hence, the missing values were multiply imputed using 100 iterations. The main characteristics of the study population are outlined in Table 1. Almost all patients were Western (95%) and had type 2 diabetes (93%).

### Psychometric quality of the PACIC and the PACIC+

#### Reliability

The internal consistency of the items within the PACIC was good. Cronbach’s alpha was 0.916 with complete case analysis and 0.919–0.920 for MI datasets. The internal consistency of the PACIC+ was comparable to the PACIC’s internal consistency. Cronbach’s alpha was 0.907 for complete cases and 0.909–0.913 for MI datasets.

#### Explorative factor analysis (EFA)

The EFA results were diffuse for the PACIC as well as for the PACIC+. The three EFA methods—PAF, PA, and Velicer’s MAP—identified varying number of factors for the PACIC and the PACIC+. The PAF, PA, and Velicer’s MAP test with the complete cases identified 4, 8, and 2 factors, respectively, for the PACIC. The PACIC+ had 5, 6, and 3 factors, respectively. EFA on the MI datasets showed comparable inconclusive results. These results imply that no specific subscales of the PACIC and the PACIC+ can be inferred with confidence.

#### Confirmatory factor analysis (CFA)

Because different factor structures were suggested by the different EFA methods, we tested all factor structures proposed by PAF-analysis if at least 2 items were included per factor. In addition, we tested the factor structure proposed by Glasgow et al. [8]. All models gave a p-value below 0.001, which implies that the null hypothesis of perfect fit is rejected. We assessed the fit using RMSEA, but none of the models had an acceptable fit (all models had a RMSEA over 0.10) (Table 2).

### Discriminant capacity between GP practices

The results of the multilevel model analysis that considered patients at first level and GP practices at second level are outlined in Table 3 for the MI data. The null model of the multilevel analysis with PACIC and PACIC+ scores showed an ICC of 0.052 and 0.051, respectively (Table 3). These results imply that 5.2% and 5.1% of the total variance of the PACIC and PACIC+ scores can be attributed to variation on GP practice level. Regarding the patient characteristics, higher age and higher education levels were associated with lower PACIC and PACIC+ scores. The models with case mix adjustment showed an ICC of 0.057 and 0.054, respectively. Complete case analysis showed no substantial differences (Appendix 3).

## Discussion

This study examined the PACIC to improve and validate its potential to measure the experience of diabetes patients in daily chronic care practice. The results revealed that the reliability of the PACIC is good to measure chronic care experience of patients with diabetes. Moreover, our results revealed that the reliability of the extended PACIC (PACIC+), which also included team functioning, was also good. No specific subscales of the PACIC and the PACIC+ can be inferred with confidence. Furthermore, the PACIC and PACIC+ identified variation in patients’ experience between GP practices.

This was the first study that assessed the psychometric quality of the PACIC by applying the appropriate statistics for the original scale structure proposed by Glasgow et al. [8]. Our results regarding the psychometric quality (reliability and no inferred subscales) are in line with the results of Gugiu which performed a validation study with another unsuccessful PACIC response category [12]. Furthermore, we could not infer a specific factor structure with confidence, which implies that previous identified subscales should not be used for quality improvement efforts. Patients chronic care management experiences scores of the PACIC should only be used as overall score or be related to one of the 26 specific items to prioritize quality improvement goals. The five factors suggested by Glasgow were only identified when we applied the inappropriate Pearson correlations that were used in earlier validation studies (data not shown). Thus, differences between our results and previous validation studies that ignored the ordinal data structure could be explained by the difference in methods. Hence, the previously incorrectly identified five-factor structure should be used with caution because of methodological limitations.

Furthermore, in addition to the study by Gugiu, we extended the PACIC by including multidisciplinary team functioning items, which are essential for the quality of chronic care based on the chronic care model [33]. This study showed good reliability scores of the PACIC+. This result implies that the PACIC+ can be of additional value without a loss of psychometric quality compared with the PACIC.

In addition to previous validation studies, this study evaluated the extent to which the variation of patients’ chronic care experience was related to the GP practices from which they receive their chronic diabetes care. The influence of this so-called ‘second level’ has only been studied once, by Glasgow and colleagues, and they identified the physician influence [10]. In line with other patient experience questionnaires, our results revealed that both the PACIC and the PACIC+ could be used to compare GP practices. Although the discriminator capacity of the GP practices can be expected to differ between settings [34], its magnitude was equivalent to or even slightly higher than previous patients experiences studies revealed [14, 34, 35]. In addition, the potential discriminator capacity is expected to be higher because we have selected a relatively homogenous group; all included GP practices already cooperate within one of the recently introduced cooperatives (i.e. care groups). In other words, the included GP practices were frontrunners regarding chronic care management at the start of our study. Future research should study the influence of this recently introduced third level, the care group, as these groups aim to improve the quality of care of their GP practices. Because the PACIC and PACIC+ were applicable as benchmark tools, our results also revealed that case-mix adjustment was needed for both instruments.

The PACIC and PACIC+ were validated with a substantial number of respondents to gain insight about a useful tool for professionals, policy-makers and researchers to assess and improve the quality of diabetes care. Nevertheless, this study had several limitations. First, we validated a disease-specific instrument to assess patients’ chronic care experience. However, it can be questioned whether this instrument should be used every year to identify the quality of chronic care for specific diseases (in this case diabetes) or if it should be transformed into a generic chronic care instrument. Second, we could not preclude social desirable answers. Though, social desirability is likely to be restricted as the first three authors sent the questionnaires directly to the patients without interferences of the GPs. The potential influence of GPs attitude or willingness to attain high patient experience scores will also be limited as results were not reported to the GP practices at GP practice level. Third, there was a relatively low response rate (46%). Analysis of non-responders was not possible, but the patient characteristics of the study population were not substantially different from those of the patients with diabetes included in the diabetes care program within the eight care groups (data not shown). In addition, differences persisted between the patients that fully completed the questionnaire and patients who had one or more missing response. Although we applied multiple imputation to address the missing data [29, 30], improvement of the questionnaire for future research would better reduce the differences in response rates regarding these patient characteristics: age, educational level, sex and co-morbidity.

Although the PACIC is already widely used, further steps to evaluate chronic care management experiences of patients are needed. First, additional qualitative research is needed to improve the response rate. This will require an understanding of why respondents did not answer certain questions in the PACIC. Second, the Dutch PACIC, and possibly also the other versions [8, 36–38], may need some improvement regarding several items that are not applicable for all patients and/or could be achieved in another way. For instance, ‘referring patients to a dietician or social worker every year’ is not necessary for all diabetic patients according to the guidelines, and ‘getting a copy of the treatment plan’ could be achieved in another way because web-based IT systems are being implemented. Because some of these changes will not be country specific [37], consensus should be achieved internationally to ensure uniformity for future comparisons between countries. Third, we extended the PACIC with six questions to incorporate CCMs team functioning component. Although these six questions are frequently applied in Dutch patient questionnaires and translated into English via the forward/backward method, validation of these translated questions could be of additional value. Fourth, the association between PACIC scores and patient outcomes needs to be studied. Because many countries shape their health system in response to chronic care model assumptions—such as the Accountable Care Organisations (ACOs) in the US, the Clinical Commissioning Groups (CCGs) in England, and Care Groups (CGs) in the Netherlands—insight into its effectiveness and patient-centred effect measures are greatly needed.

## Conclusions

The PACIC and the PACIC+ are both reliable instruments to measure the chronic care management experiences of patients with diabetes. Previously identified subscales should be used with caution. The PACIC+ should be preferentially used because it also includes multidisciplinary team functioning, which is one of the central pillars of chronic care management. Both PACIC instruments can be used to identify variation in chronic care management between GP practices. Nevertheless, it is advisable to further improve the PACIC+ to increase the content validity and response rates.

## Acknowledgement

JTJ, JNS, CAB and HWD contributed to the conception and design of the questionnaires. JTJ collected and imported all data. HWD analyzed the data and wrote the article. All authors contributed to the design of the study, reviewed and edited the manuscript, and approved the manuscript.

H.W. Drewes MSC is the guarantor of this work and, as such, had full access to all of the data in the study and takes responsibility for the integrity of the data and the accuracy of the data analysis.

The authors wish to thank care groups’ managers and all patients who participated in this study. The authors wish to extend their gratitude to M. Schipper PHD of the National Institute for Public Health and the Environment for useful comments regarding multiple imputations in R.

## Reviewers

**Chris Coryn,** Director of the Interdisciplinary, PhD in Evaluation, Associate Professor of Evaluation, Measurement, and Research, Western Michigan University, USA

**K. Viktoria Stein,** Institute of Social Medicine, Centre for Public Health, Medical University of Vienna, Austria

**H.J.M. (Bert) Vrijhoef,** PhD, Professor Chronic Care, Tilburg University, Scientific Center for Care and Welfare (TRANZO), The Netherlands

## Figures and Tables

**Table 1.**
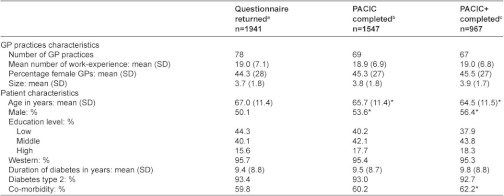
Patient characteristics

a) Patients who returned the questionnaire, not all patients completed the questionnaire fully; b) patients who completed the PACIC without missing responses; c) patients who completed the PACIC+ without missing responses; *Significant difference compared to patients that returned the questionnaire.

**Table 2.**
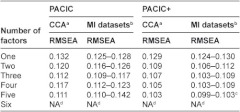
Results of CFA with factor structure proposed by PAF analysis

a) CCA, complete case analysis; b) MI datasets, analysis of the first 5 multiple imputated datasets; c) RMSEA<0.10 in only one MI dataset; d) NA, not assessed since the number of variables in one factor was <2.

**Table 3.**
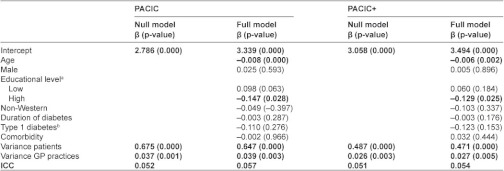
Multilevel analysis results with multiple imputed data

a) Reference is middle education level; b) reference is type 2 diabetes, ICC, intra-class correlation.
